# Cough Radiculopathy: Postinfectious Cough-Related Acute Lumbar Radiculopathy

**DOI:** 10.1155/2020/2360854

**Published:** 2020-08-13

**Authors:** Samer Alkhuja

**Affiliations:** The Commonwealth Medical College, Lehigh Valley Health Network-Pocono, 1655 West Main Street, Stroudsburg, PA 18301, USA

## Abstract

Cough is a protective reflex of airways and lungs. Cough may result in several complications. Postinfectious cough is a cough that begins during an acute respiratory tract infection, usually is self-limited, and is due to airway hyperresponsiveness as a result of airway inflammation. Cough radiculopathy has been once reported by Torrington and Adornato in the form of acute cervical radiculopathy. This is a case of acute lumbar radiculopathy as a result of postinfectious cough. Literature review did not show a similar case.

## 1. Introduction

Cough is a protective reflex of airways and lungs. Cough may impact patient's quality of life [1] and result in several complications [2] (Table 1). Postinfectious cough (PIC) is a cough that begins during an acute respiratory tract infection, lasts more than 3 weeks but less than 8 weeks, is not complicated by pneumonia, and usually is self-limited [3]. PIC may be due to airway hyperresponsiveness as a result of airway inflammation [1, 3, 4]. Chest radiograph (CXR) findings are usually normal [3].

Cough radiculopathy (CR) has been once reported by Torrington and Adornato in the form of acute cervical radiculopathy [5]. This is a case of acute lumbar radiculopathy as a result of PIC. Literature review did not show a similar case.

## 2. Case

A 60-year-old man developed a PIC which started four weeks after recovering from an upper respiratory tract infection. The violent PIC resulted in the development of abrupt onset lumbar radiculopathy with progressive muscle weakness. There was no loss of bladder or bowel control. The patient had weakness that resulted in inability to ambulate independently. The patient reported no history of trauma, allergies, tobacco or recreational substance use, or alcohol abuse. There was evidence of sciatica and dysesthesia radiating to the left lower extremity in the lumbar (L) L3-L4 dermatome.

Past medical history included coronary artery disease, hypertension, hyperlipidemia, erectile dysfunction, colitis, diabetes, and gastroesophageal reflux disease. Past surgical history included bilateral inguinal hernia repair and cholecystectomy. Current medications were ranolazine, tadalafil, prasugrel, omega-3 fatty acids, vitamin D3, lisinopril, budesonide, rosuvastatin, pantoprazole, multivitamin, aspirin, nebivolol, ezetimibe, and famotidine.

Physical examination showed left lower extremity weakness with positive straight-leg sign. Babinski sign showed a downward response. Motor system examination revealed generalized normal bulk and tone except in the left lower extremity. The power was 3/5 at the left knee and left foot. Deep tendon reflexes (DTR) were +2 in upper and right lower extremities and zero at the left knee and ankle. Sensory system examination showed intact pinprick and light touch in all extremities except for a symmetrical decrease distally in feet. Gait testing was limited due to left lower extremity. Romberg's test was negative. Coordination showed intact finger-to-nose and heel-to-shin tests.

Magnetic resonance imaging (MRI) of the lumbar spine showed no spinal or foraminal stenosis at L1-2, L2-3, L4-5, or L5-S1. At L3-L4, there was superiorly extruded left paracentral disc herniation. There was evidence of facet degenerative changes.

There was no abnormal epidural or dorsal nerve root enhancement.

CXR, pulmonary function test, complete blood count, serum electrolytes, and liver function tests were all normal.

Medical treatment included antitussive medications, acetaminophen, cyclobenzaprine, and oral corticosteroids. Symptoms persist; therefore, left hemilaminectomy at L3-L4 was performed, which resulted in the recovery of neuromuscular deficits.

## 3. Discussion

### 3.1. Postinfectious Cough

In nasal airways, an URTI induces neuromodulation which contributes to the hyperreactivity in the airway and cough. In addition, the neuromodulation may outlive the infection and cause persistent symptoms, which explains the persistence of PIC symptoms well beyond the time needed for immunological clearance of the infection [6]. Binding of a virus to the intercellular adhesion molecule I on the airway cell triggers the recruitment of cells and the release of inflammatory cytokines [6], which upregulates the cough reflex [6].

### 3.2. Radiculopathy

Back pain is the fourth leading cause of disability in the world [7]. Nearly 80% of the population sustains an episode of low back pain once during their lifetime [7]. Some patients with lumbar disc herniation (LDH) may have evidence of severe degenerative disc changes. Disc herniation occurs as a result of axial overloading [7, 8]. The overloading could be static or dynamic [7, 8]. During abrupt dynamic overloading, pressure shifts from the center towards the periphery of the intervertebral disc which results in disc herniation (Table 1) [2, 8]. This mechanism explains the development of vertebral disc herniation that results from PIC [8]. An individual may have asymptomatic degenerative spondylosis changes until a sufficient stimulus, such as “violent cough,” brings out the symptoms or causes acute radiculopathy (this patient) [5].Violent cough can lead to nerve root complications rather than a causal relationship.

In 2014, the Work Group of the North America Spine Society's Evidence-based guideline Development Committee recommended manual muscle testing, sensory testing, and spine straight-leg raising (SLR) test as the gold standard tests for the clinical diagnosis of LDH [9]. Initial screening by the SLR test in conjunction with three of the following four symptoms in a nerve root distribution is sufficient for clinical diagnosis of LDH with radiculopathy: dermatomal pain, sensory deficits, reflex deficits, and/or motor weakness [7, 9, 10].

## 4. Radiographic Studies

Radiographs: plain radiographs are the first-line imaging modality used in low back pain [7, 10]. MRI is the gold standard for imaging to confirm LDH with a diagnostic accuracy of 97% [7, 10]. Diffusion tensor imaging is a type of an MRI that can be used to detect microstructural changes in the nerve roots in patients with LDH [7, 10]. CT scan advances including the multidetector CT scan have brought the diagnostic level of CT to be nearly equal to that of an MRI [7, 10]. Finally, in certain circumstances, a CT myelography would be chosen as opposed to other imaging modalities [7, 10].

Since both CT scan and MRI studies are characterized by high prevalence of abnormal findings even in asymptomatic individuals, performing these studies should be considered only for cases involving focal neurologic symptoms or when there is a need for interventional treatment [7, 10].

## 5. Treatment

### 5.1. Postinfectious Cough

 It may respond to dextromethorphan, oxatomide, antihistamine, bakumondoto (a Japanese medicine controlling activities of C fibers in patients suffering from constant coughs after upper airway infections), montelukast, nasal spray (ipratropium bromide) and nasal corticosteroids, and a short-term oral prednisone treatment [3].

### 5.2. Radiculopathy

In the absence of myelopathy or muscle weakness, all patients should be treated conservatively for at least six weeks [7, 10, 11]. Conservative treatments consist of immobilization, physical therapy, cervical traction, the use of muscle relaxants and anti-inflammatory medications, and education [7, 10, 11].

Surgical approach should be considered if conservative treatments failed or those who have significant findings of radiculopathy, muscle weakness, or myelopathy [12].

## 6. Conclusion

Severe coughing can lead to nerve root complications such as acute cervical (Table 1) [2, 5] or lumbar radiculopathies (this case) rather than a causal relationship. Treating violent cough should be aggressive, especially in patients who have a known pre-existing degenerative spondylosis, to prevent the development of symptomatic radiculopathy. Abrupt cough may result in dynamic overloading and pressure shifting from the center towards the periphery of the intervertebral disc [8], which explains the mechanism of abrupt disc herniation in PIC.

## Figures and Tables

**Table 1:** Complications of excessive cough (Irwin, “Complications of cough: ACCP evidence-based clinical practice guidelines,” [2].

Respiratory	Subcutaneous emphysema, pneumomediastinum, pneumothorax, exacerbation of asthma, laryngeal trauma
Cardiovascular	Arterial hypotension, dislodgment/malfunctioning of intravascular catheters, bradyarrhythmias, and tachyarrhythmias
Gastrointestinal	Gastroesophageal reflux events, Mallory–Weiss tear, splenic rupture, herniation
Neurological	Headache, dizziness, cough syncope, cerebral air embolism, acute cervical radiculopathy, seizures, and stroke due to vertebral artery dissection
Genitourinary	Urinary incontinence, inversion of the bladder through the urethra
Musculoskeletal	Rib fractures, diaphragmatic rupture
Others	Fear of serious disease, decreased quality of life, disruption of surgical wounds, petechiae, and purpura
